# A West Nile Virus infection expressed as unilateral limb paralysis and complicated by Parsonage–Turner syndrome: a case report

**DOI:** 10.1186/s13256-023-03756-w

**Published:** 2023-02-15

**Authors:** Antonella Scarciglia, Luca Roncucci, Piero Benatti

**Affiliations:** 1 School of Emergency Medicine, University of Modena and Reggio Emilia, Modena, Italy; 2 Department of Maternal, Child and Adult Medical and Surgical Sciences, University of Modena and Reggio Emilia, Modena, Italy

**Keywords:** Case report, West Nile Virus, Parsonage–Turner syndrome, Flaccid paralysis, Arbovirus

## Abstract

**Background:**

West Nile Virus is a single-stranded Ribonucleic Acid arbovirus of the Flaviviridae family that is transmitted to humans by *Culex* species mosquitoes. West Nile Virus infection is asymptomatic in the majority of affected people. Of those who develop symptoms, the usual manifestation is a febrile syndrome, while only 1% develop neurological symptoms due to a neuroinvasive form of infection, including encephalitis, meningitis, asymmetrical flaccid paralysis, or a combination of all these features. Parsonage–Turner syndrome is a rare disorder characterized by sudden painful symptoms and subsequent paralysis, involving a shoulder or one of the upper limbs due to post-infective brachial plexopathy. The etiology is unknown, although it can be considered a multifactorial process: a predisposing factor, such as viral infection or strenuous upper-extremity exercise, can trigger an immune-mediated process localized in the brachial plexus.

**Clinical presentation:**

In late summer, a 79-year-old male Italian patient was admitted to the emergency department for acute right upper limb weakness and high fever, without any mental status impairment, pain, sensory alterations, or signs of meningeal irritation. Laboratory tests confirmed acute West Nile Virus infection, expressed as a unilateral upper limb flaccid paralysis. After a few days, the patient reported an acute pain in the right upper limb scarcely responsive to nonsteroidal anti-inflammatory drug therapy and a subsequent wider distribution of flaccid paralysis. After multiple examinations, Parsonage–Turner syndrome could be suspected. Patient was treated with steroids and reported an improvement of clinical condition after 2 months, with complete pain remission but partial strength recovery in the affected limb.

**Conclusions:**

West Nile Virus disease has a broad spectrum of neurological manifestations, among which the most common are signs of meningeal irritation or cognitive impairment. We report an unusual presentation of neuroinvasive West Nile Virus infection with arm weakness as expression of unilateral viral neuritis, followed by a post-infective brachial plexopathy consistent with Parsonage–Turner syndrome diagnosis. We diagnosed Parsonage–Turner syndrome after excluding the most common causes of atraumatic acute upper limb pain, through a challenging differential diagnosis in a patient with several comorbidities.

## Background

West Nile virus is a single-stranded Ribonucleic Acid (RNA) arbovirus of the Flaviviridae family, transmitted to humans by *Culex* species mosquitoes [1]. The incubation period is 2–14 days, after which 80% of affected people do not develop any symptoms. Of those who develop symptoms, the usual manifestation is a febrile syndrome. Only 1% of symptomatic patients develop neurological symptoms due to a neuroinvasive form of infection, including encephalitis, meningitis, poliomyelitis-like syndrome, or an overlap of all these features [2]. Poliomyelitis-like syndrome is due to selective destruction by West Nile virus of motor neurons of the spinal cord, resulting in acute flaccid paresis or paralysis [3]. There is no specific treatment for West Nile virus infection, so treatment is based on supportive care, such as antipyretic drugs or intravenous fluid therapy. Parsonage–Turner syndrome is a rare disorder characterized by sudden pain and subsequent paralysis, involving one of the upper limbs, due to brachial plexopathy [4]. The etiology is unknown, although it can be considered a multifactorial process: a predisposing factor, such as viral infection or strenuous upper-extremity exercise, can trigger an immune-mediated process localized in the brachial plexus [5]. Parsonage–Turner syndrome is associated with good prognosis, but it has a long recovery period. Corticosteroids are usually effective in facilitating strength recovery and reducing the need for pain reliever medications.

We report an unusual case of neuroinvasive West Nile virus infection with arm weakness as expression of viral neuritis, followed by a post-infective brachial plexopathy consistent with Parsonage–Turner syndrome diagnosis.

## Case presentation

A 79-year-old male Italian patient living in a rural area was admitted to our emergency department for acute right upper limb weakness, associated with high fever (39 °C). The fever had initiated 10 days before and had been treated at home with acetaminophen and antibiotic therapy (amoxicillin/clavulanic acid for 4 days, then levofloxacin plus ceftriaxone for 2 days). There was no history of trauma or recent travel. The medical history of the patient included type II diabetes, arterial hypertension, and hypothyroidism. At the time of admission, the patient was febrile (37.8 °C), tachypneic (30 respiratory acts per minute), and with elevated blood pressure (190/100 mmHg). General examination revealed dehydration signs, whereas neurological examination showed localized muscle weakness: 2 out of 5 on Medical Research Council scale in the triceps, extensors and flexors of wrist, and fingers; 3 out of 5 in the biceps, abductors of the arm, flexors of the arm, and forearm. The patient did not show altered mental status, pain or sensory alterations, or signs of meningeal irritation.

Sample blood tests were unremarkable, with normal inflammatory indices. Blood and urine cultures were found to be negative. Brain computed tomography scan was unremarkable, as was brain magnetic resonance imaging, which excluded parenchymal infectious-related lesions, white matter lesions, or meningeal lesions. Chest computed tomography excluded embolism, pneumonia, and pleural and pericardial effusion. Abdominal ultrasound did not show pathological features.

A new anamnestic interview was performed, and the patient revealed several mosquito bites at home, leading us to hypothesize arbovirosis. A positivity of serum Immunoglobulins (Ig)M, IgG, and RNA polymerase chain reaction for West Nile virus was detected, leading to the diagnosis of West Nile virus infection, according to Centers for Disease Control and Prevention (CDC) criteria [1].

After spontaneous resolution of fever, 4 days after admission the patient reported an acute right upper limb pain that worsened at night and was not modified by rest or activity. After about 24 hours, neurological re-examination showed a wider distribution of muscle weakness, also involving interosseous, opponens pollicis, and opponens digiti minimi muscles (2 out of 5 on Medical Research Council scale) and trapezius (3 out of 5 on Medical Research Council scale). A new sensory evaluation was not performed. Moreover, shoulder ultrasound showed supra- and infraspinatus tendinosis with insertional calcifications and full-thickness rupture of the subscapularis tendon. Magnetic resonance imaging of the spine showed posterior disc protrusion at C7–C8 right level. Nerve conduction study and electromyography revealed three types of lesions: symmetric mixed sensorimotor polyneuropathy, mainly extended to the lower limbs; cervical polyradiculopathy, predominant at right C7–C8 level; signs of recent nerve damage at right C5–C7 level, with preservation of sensory nerve potentials.

We diagnosed Parsonage–Turner syndrome by excluding the most common causes of nontraumatic upper limb acute pain. Supra- and infraspinatus tendinosis and rupture of the subscapularis tendon were inconsistent with the clinical manifestation and the physical examination of the patient: in particular, pain was not exacerbated by active or passive movements or by specific orthopedic tests, such as Gerber’s lift-off test. Nerve conduction findings could be explained by three different types of lesions: lower limb mixed polyneuropathy was compatible with diabetic polyneuropathy; cervical radiculopathy at C–C8 level with cervical disc protrusion; right cervical polyradiculopathy at C5–C7 level with West Nile virus neural damage. However, the upper limb pain was not fully explained by the diagnosis of cervical herniation because it did not match C7–C8 dermatome distribution and it was not exacerbated by neck movements or Spurling’s maneuver. Other causes, such as adhesive capsulitis, were excluded because the severe acute pain was associated with weakness, rather than stiffness of the shoulder. Furthermore, the localization of pain, which mainly involved the shoulder, did not correspond with the nerve territory distribution of the paresis, which involved extensively the right upper limb. This feature is typical of Parsonage–Turner syndrome [5].

On this basis, we diagnosed a post-infective brachial plexopathy, also known as Parsonage–Turner syndrome.

A therapy with oral prednisone (25 mg twice daily) and tapentadol (50 mg twice daily) was set up. A dermal patch of lidocaine (700 mg, 5% w/w) was applied as a local anesthetic. The patient was recommended to avoid strenuous activities and to rest for a few weeks.

After 4 weeks of analgesic medications and corticosteroid treatment, upper limb pain progressively disappeared, while hyposthenia partially recovered. After 2 years, the patient was still pain-free and his degree of arm motion was conditioned only by the rotator cuff injury.

## Discussion and conclusions

West Nile virus is a single-stranded RNA arbovirus of the Flaviviridae family. It is transmitted to humans by *Culex* species mosquitoes, while birds act as amplifying reservoir hosts [6]. It appeared for the first time in 1937 in Uganda (in the West Nile district), then largely spread through Africa, Western Asia, Australia, America, and Europe [7, 8]. Its first appearance in Italy was in the region of Tuscany in 1998. Since then, it has spread through the country, especially in the swampiest areas [9]. Most West Nile virus infections in Europe generally occur between July and October, when mosquito abundance is highest [10]. After an incubation period of 2–14 days, only 20–40% of infected subjects show clinically relevant manifestations. Most cases develop fever, while only 1% develop neurological symptoms due to a neuroinvasive form of infection, including encephalitis, meningitis, asymmetrical flaccid paralysis, or a combination of all these features. Acute paralysis results from destruction of the anterior horn cells of the spinal cord, and it is characterized by acute onset (over 48 hours) of limb asymmetrical weakness with hyporeflexia and absence of pain or sensory abnormalities. According to CDC diagnostic criteria, the diagnosis of West Nile virus infection is based on specific laboratory tests, such as IgM and IgG enzyme-linked immunosorbent assays (ELISAs) and viral RNA detection via real time polymerase chain reaction (RT-PCR). There is currently no specific treatment nor human vaccine for West Nile virus disease. Patients can be treated with only supportive therapies, such as intravenous fluids or antipyretics drugs. Prevention of infection by protection from mosquito bites is the most effective strategy for reduction of illness [11]. The case fatality rate in Europe is usually 12% [10]*.*

Parsonage–Turner syndrome, also known as neuralgic amyotrophy, is a rare brachial plexopathy characterized by acute intense upper limb pain and subsequent progressive flaccid paralysis and muscle damage without specific nerve root distribution [12]. Pain is not positional and typically worsens at night.

Hypoesthesia or other sensory signs are usually mild, and they are observed in 80% of patients. Parsonage–Turner syndrome is more common in men than in women. Greater involvement of the dominant limb, which is more frequently the right, has been observed. Bilateral brachial plexus involvement is unusual, but possible [13]. The cause is unknown, but the leading hypothesis is an immune-mediated reaction to an environmental factor, such as viral infection, vaccination, intense upper-limb exercise, pregnancy or puerperium, and surgery. Other hypotheses consider the nervous impairment resulting from the direct action of the virus or from local ischemia that causes blood–nerve barrier damage [5]. Constitutional mutations in *SEPT9* gene located on chromosome 17q cause the hereditary form of Parsonage–Turner syndrome, characterized by recurrent attacks and positive family history [14]. The diagnosis is clinical. Routine blood tests are usually normal, without elevation of inflammatory markers. Electromyography can support the diagnosis by showing denervation signs, but it is not sensitive to the high risk of false negatives due to sampling errors. Imaging studies are useful to exclude other differential diagnosis. The pain usually responds to the combination of long-acting opioids with nonsteroidal anti-inflammatory drugs [13]. Current data on the efficacy of prednisolone in pain control are conflicting, though it seems to be effective in decreasing the duration of pain and facilitating full strength recovery [15]. There is no evidence of the usefulness of immunomodulation treatment. The prognosis is good: pain usually disappears in 3 weeks, while peripheral nerve function is restored in 2–3 years in 80–90% of patients. Intense exercise is not recommended because it can delay the reinnervation process [16].

On review of the literature, the association between West Nile virus and Parsonage–Turner syndrome is reported in only a few cases. Almhanna *et al*. [17] and then Scholz *et al*. [18] described brachial plexitis associated with West Nile virus disease, with plexopathy preceding meningoencephalitis, the classical form of neuroinvasive infection. Chahil *et al*. [19] reported a case of subacute brachial plexopathy associated with West Nile virus infection, not consistent with the diagnosis of Parsonage–Turner syndrome. To our knowledge, this is the first description of a West Nile virus infection causing asymmetric flaccid paralysis without any cerebral or meningeal involvement, followed by Parsonage–Turner syndrome as a complication. This case report has some limitations: as Parsonage–Turner syndrome is diagnosed on only a clinical basis, there is no diagnostic test that can confirm our speculation. Moreover, when the patient reported the acute shoulder pain, a second sensitivity evaluation was not performed: we may have missed a mild sensitivity impairment, which is often observed in Parsonage–Turner syndrome. However, owing to the complexity of the clinical presentation, the patient has been examined by a multidisciplinary team of experts: orthopedic surgeons, neurologists, neuroradiologists, infectious disease specialists, and internal medicine physicians. The final diagnosis has been made thanks to cooperation and consensus of all these specialists. Another strong point is the clinical improvement of the patient with corticosteroid therapy.

We suspected West Nile virus infection expressed by fever and motor neuropathy thanks to an accurate anamnestic interview. We also diagnosed Parsonage–Turner syndrome after excluding the most common causes of atraumatic acute upper limb pain, through a challenging differential diagnosis in an elderly patient with several comorbidities. This emphasizes the importance of detailed anamnesis and accurate clinical examination, which are still very important in guiding the physician towards the right diagnosis. The present case shows a rare occurrence of an atypical presentation of West Nile virus infection associated with Parsonage–Turner syndrome. Further studies are needed to explore the pathogenesis of this association and the optimal therapeutic management.

## Data Availability

Not applicable.
